# High Levels of Proinflammatory Cytokines, but Not Markers of Tissue Injury, in Unaffected Intestinal Areas from Patients with IBD

**DOI:** 10.1155/2009/580450

**Published:** 2009-07-30

**Authors:** Alberto J. León, Emma Gómez, Jose A. Garrote, David Bernardo, Asterio Barrera, Jose L. Marcos, Luis Fernández-Salazar, Benito Velayos, Alfredo Blanco-Quirós, Eduardo Arranz

**Affiliations:** ^1^Mucosal Immunology Laboratory, Department of Pediatrics & Immunology, IBGM, University of Valladolid, 47005 Valladolid, Spain; ^2^Division of Immunology, International Institute of Infection and Immunity, Shantou University, Guangdong 515041, China; ^3^Research Unit, Hospital Clínico Universitario, 47011 Valladolid, Spain; ^4^General Surgery Unit, Hospital Universitario Rio-Hortega, 47012 Valladolid, Spain; ^5^Gastroenterology Unit, Hospital Clínico Universitario, 47011 Valladolid, Spain

## Abstract

Intestinal alterations in IBD are triggered and maintained by an overexpression of proinflammatory cytokines. Additionally, increased immune activation has been found in the adjacent intestinal areas without displaying any apparent histological alterations, however, the regulatory environment is not well established. Biopsy specimens from patients with ulcerative colitis (UC) and Crohn's disease (CD), from both affected and unaffected areas, and also from a group of colonic biopsies from healthy controls, were included in our study. Cytokines and markers of mucosal damage were analyzed by real-time PCR, and some of the results confirmed by western-blot and ELISA. Levels of IFN*γ*, TNF*α*, IL-6, IL-15, IL-18, and IL-23 were increased (above healthy controls) in both affected and unaffected areas from IBD. IL-1*β*, IL-6, IL-12, and IL-27 were higher in affected areas compared to unaffected ones in UC but not CD. In general, a correlation was observed between mRNA levels of these cytokines and both iNOS and Granzyme B. SOCS-2 and SOCS-3 were also increased in the affected areas. In conclusion, the unaffected areas from IBD show increased levels of a restricted set of cytokines that may exert immune activating roles in these areas without being able to trigger tissue damage.

## 1. Introduction

Ulcerative colitis (UC) and Crohn's disease (CD) are severe intestinal inflammatory conditions which result from a dysregulated proinflammatory response to components of the gut microflora, inadequately controlled by normal regulatory/homeostatic mechanisms [1]. The expression of proinflammatory cytokines is increased in inflammed biopsy areas from both UC and CD, and the effect of these cytokines on the intestine has been directly related with the induction of tissue injury (destruction) [2, 3]. 

Most of the reports published so far on these chronic inflammatory diseases are mainly focused on the pathological events occurring in areas of the intestine with a histologically altered mucosa. However, there is evidence suggesting that the unaffected tissue areas of IBD undergo an abnormal immune activation as shown by the expression of increased levels of the proinflammatory cytokines IL-6, TNF*α* [4], and IL-18, in intestinal biopsies from CD patients [5]. Contrary to CD, where the involvement of unaffected areas is better known, further studies are needed to confirm the presence of inflammatory mediators in the unaffected tissue areas from UC. 

The increase in proinflammatory cytokines and markers of mucosal damage in the affected areas of IBD is wellestablished. Different effector mechanisms may be activated during intestinal inflammation, such as inducible nitric oxide synthase (iNOS), a key inflammatory mediator which increases NO levels [6] and is involved in the control of mucosal damage [7, 8]; the apoptosis-related molecule Granzyme B (GZNB) [9, 10]; the extracellular matrix-remodelling molecule, MMP-3 and its physiological inhibitor TIMP-1 [11]. However, little is known about which cytokines induce these effector mechanisms in the affected areas of IBD. Additionally, the regulatory activities of the immune response, through mediators such as IL-10 and TGF*β*, still need to be profiled, especially those that might take place in the unaffected areas. Moreover, the SOCS-mediated intracellular suppressor activity exerts a crucial negative feedback of the cytokine signaling, and it may play a relevant role in the control of the mucosal damage in IBD [12].

Our aims were ( 1) to study the expression of cytokines and markers of tissue damage in affected and unaffected tissue areas from IBD, ( 2) to correlate cytokine profiles to potential activation mechanisms of mucosal damage in the intestine from these chronic inflammatory diseases, and ( 3) to determine the potential role of different regulatory mediators, including certain cytokines and SOCS family members, in the preservation of histological integrity in unaffected tissue areas. 

## 2. Materials and Methods

### 2.1. Study Subjects

Patients attended the Adult Gastroenterology Clinics, from the Hospital Clínico Universitario and Hospital Universitario Río-Hortega of Valladolid (Spain), as part of the routine diagnostic procedures for suspicion of IBD, during years 2003 to 2005. Intestinal biopsies were obtained during colonoscopy using a fiberscope with forceps (Olympus, Tokyo, Japan). Patients' diagnosis was initially based on the endoscopic findings and later confirmed by histopathological analysis of biopsies taken from affected areas. The unaffected areas from which biopsies were collected were defined by endoscopic evaluation histopathological analysis of these biopsies could not be performed due to the limited amount of tissue that was collected, which required to be totally disrupted for RNA/protein purification. The Montreal classification [13] was used to define the clinical features of patients. Informed consent was obtained from patients, and the study protocol was approved by the local Ethics Committee (University Hospital, Faculty of Medicine).

Two intestinal biopsy specimens were collected from patients with IBD, one from affected areas and a second one from areas without endoscopic alterations. Study groups were composed as follows (Table 1): UC from affected (*n* = 26) and unaffected areas (*n* = 22), CD from affected (*n* = 16) and unaffected (*n* = 15), and a group of healthy controls (*n* = 16), from patients submitted to the hospital for screening or followup of other colon diseases (mostly colon cancer), but not presenting signs of mucosal inflammation or disease. 

The group of patients with UC (26 cases) has an average age of disease onset of 42.3 years, treatment was 5-ASA (14 cases), corticoids (2 cases), and untreated (10 cases). The degree of mucosal damage in the affected areas of UC was mild (5 cases), moderate (9 cases), and severe (12 cases).

The group of CD patients (16 cases) has an average age of disease onset of 37.1 years, they showed the following presentations: penetrating (3 cases), stricturing (2 cases), and nonpenetrating and nonstricturing (formerly inflammatory presentation, 10 cases). At the time of biopsy collection, 4 cases were untreated and none of the patients was receiving immunouppressors (detailed description on the treatments is shown in Table 1).

### 2.2. Sample Preparation

After collection, tissue samples were immediately submerged in 1mL of RNA-Later solution (Ambion, Austin, TX, USA) and stored at −20°C. Both total RNA and protein samples were isolated from biopsies using TRIZOL reagent following manufacturer's instructions. Protein samples were diluted in SDS 1%, and total protein concentration was determined using the DC Protein Assay (BioRad Laboratories, Hercules, CA, USA).

### 2.3. Quantitative Polymerase Chain Reaction

Quantification of mRNA expression in intestinal biopsies was performed as previously described [14]. Briefly, reverse transcription was carried out by using the SuperScript First-Strand Synthesis System for reverse transcriptase (RT)-PCR Kit (Invitrogen SA, Barcelona, Spain) using OligodT primers and mRNA levels were determined by real-time PCR using a LightCycler instrument (Roche Applied Science, Mannheim, Germany). Reactions were performed in a volume of 20 *μ*L using either FastStart SYBR Green MasterMix (Roche) or FastStart HybProbes MasterMix (Roche) for Taqman probes, and 1 *μ*L of thermolabile Uracil DNA Glycosylase (UDG) (Roche) to prevent carry-over contamination. Cytokine primer sets are described in Table 2. Samples were analyzed in triplicate and results, obtained as a ratio cytokine/*β*-actin mRNA levels, were expressed as arbitrary units.

A standard curve for each gene was made using serial dilutions from the RNA extracted from cell lines, either Jurkatt (6 × 10^6^ cells) or Thp1 (10 × 10^6^ cells), previously stimulated with 10 nM of PMA and 3 uM of Ionomycin for 5 hours.

### 2.4. Western-Blot Analysis

In all cases, 10 *μ*g of protein was added per well, and separated by using a 15% acrilamide/bisacrilamide (37.5 : 1) gel in a mini-Protean II (BioRad), and transferred onto PVDF membranes of 0.45 Micron (Pierce Biotechnology, Rockford, IL, USA). Membranes were incubated with primary antibodies to human IL-15 (mouse monoclonal MAB247, R&D, Minneapolis, MN, USA) or IL18 (mouse monoclonal SC-13602, Santa Cruz Biotechnology, Santa Cruz, CA, USA) at a final dilution of 1/400 and 1/200, respectively; a second incubation with antibodies to mouse IgG labelled with horseradish peroxidase (Amersham Biosciences Europe, Freiburg, Germany), using the chemiluminiscent substrate ECL Plus (Amersham) and the autoradiography film Hyperfilm ECL (Amersham). Finally, the QuantityOne software (BioRad) was used for molecular weight and densitometric analysis of the bands. Recombinant human IL-15 (Peprotech, London, UK) and IL-18 (active form) (Chemicon, Temecula CA, USA) were used as positive controls.

### 2.5. Protein Levels Analyzed by ELISA

To avoid interference, SDS was removed from each protein extract with the SDS-out reagent (Pierce), and the final protein concentration was determined by using the DC Protein Assay (BioRad). IFN*γ* and TGF*β* were measured in 50 *μ*g of the protein extract from biopsies, using specific Easy Elisa (Amersham) tests. Finally, the absorbance was measured by a Sunrise ELISA plate reader (Tecan, Zurich, Switzerland).

### 2.6. Statistical Analysis

Results are expressed as the median value and the interquartile range. Statistical differences in cytokine mRNA expression levels between groups were analyzed using the Mann-Whitney *U*-test, and confirmed with the Kruskal Wallis test with Dunn's posttest. Both tests showed almost identical conclusions (Table 3), the results of this paper are based on the first one. The analysis of nonparametric correlation was performed by using the Spearman's rank correlation test. A value of *P* < .05 was considered statistically significant. The statistical analysis was performed by using the software program SPSS-16.0.

## 3. Results

### 3.1. Mediators of Tissue Injury (iNOS, GZMB, and MMP-3) Are Upregulated in Affected Areas of IBD

**Figure 1:**
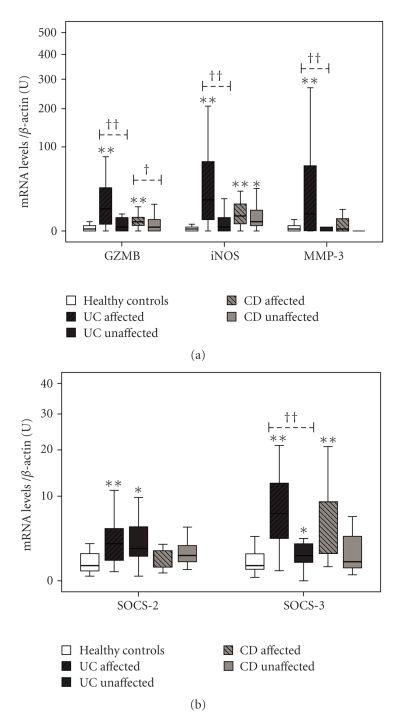
Differences in mRNA levels between study groups, (a) tissue injury-related molecules, (b) SOCS family members. Statistical differences when compared to healthy controls *P < .05 and **P < .001, and between affected and unaffected areas from the same pathology ^††^ *P* < .001. U: arbitrary units.

In biopsies from patients with UC, both MMP-3 and TIMP-1 mRNA levels are increased in affected areas, as compared to healthy controls (*P* = .018 and *P* = .001, resp.) (Figure 1), whereas unaffected showed no changes. Moreover, iNOS and GZMB were also increased at the mRNA level in affected (*P* < .001 and *P* = .006, resp.), but not unaffected areas. In biopsies from patients with CD, mRNA levels of iNOS are increased above healthy controls in both affected (*P* < .001) and unaffected areas (*P* = .019), while GZMB was increased only in affected areas (*P* = .009). TIMP-1 mRNA levels were also raised in both affected (*P* = .043) and unaffected areas (*P* = .045), from CD patients. No changes in the levels of MMP-3 were found in CD. When comparing mRNA levels between affected areas of UC and CD, iNOS, GZMB and MMP-3 show higher levels in UC (*P* = .036, .037 and .014, resp.).

No significant correlations between mRNA levels of molecules involved in tissue damage (iNOS, GZMB, or MMP-3), and the histological score of mucosal damage of the affected areas were found in either UC or CD, moreover, cytokine mRNA levels did not correlate with the histological score. There were no significant findings when comparing groups of samples according to their clinical presentation of CD or the treatment, probably due to the small amount of samples on each subgroup (data not shown).

### 3.2. Proinflammatory Cytokines (IFN*γ*, TNF*α*, IL-6, IL-15, IL-18 and, IL-23) Are Increased in Both Affected and Unaffected Areas

**Figure 2:**
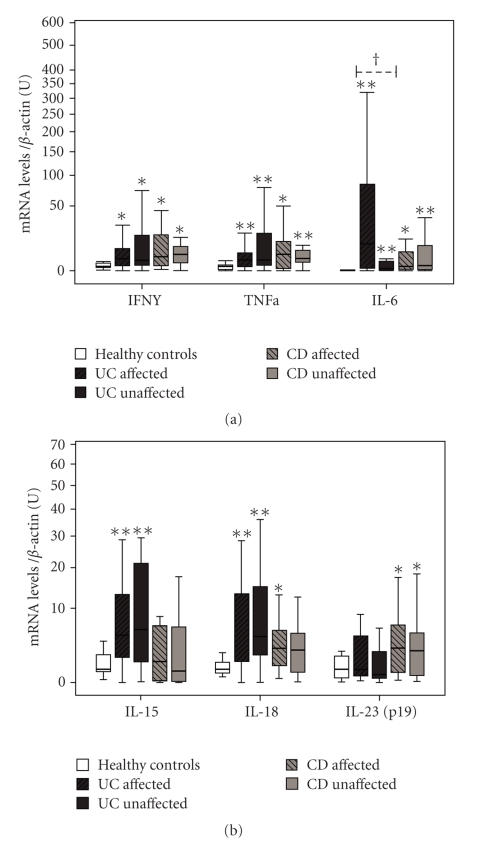
Differences in mRNA levels of cytokines increased only in both affected and unaffected areas of IBD. Statistical differences compared to healthy controls *P < .05 and **P < .001, and between affected and unaffected areas from the same pathology ^†^ *P* < .05. U: arbitrary units.

IFN*γ* and TNF*α* mRNA levels are increased above healthy controls in UC from affected (*P* = .046 and *P* = .005, resp.) and unaffected areas (*P* = .038 and *P* = .004) as compared to healthy controls (Figure 2). Also in biopsies from CD patients, IFN*γ* and TNF*α* mRNA levels were significantly increased in affected areas (*P* = .024 and *P* = .029) and unaffected areas (*P* = .027 and *P* = .004). Protein levels of IFN*γ* measured by ELISA were found to be increased above healthy controls (4.350[3.075]) in both affected (10.000[5.938], *P* = .033) and unaffected areas (8.850[6.887], *P* = .041) from UC, whereas no significant differences were found in CD (data not shown). 

IL-6 levels are increased in both affected and unaffected areas (*P* < .001 and *P* = .002, res.) from UC, and also in affected and unaffected samples from CD patients (*P* = .010 and 0.004). Increased IL-15 mRNA levels were found in both affected (*P* = .003) and unaffected areas (*P* = .001) from UC, compared to healthy controls, but not in CD. At the protein level (Figure 4), IL-15 expression was found in affected (15 out of 26) and unaffected areas (11 out of 22) from UC, as well as in affected (6 out of 16) and unaffected areas (7 out of 15) from CD, but in none of the healthy controls. IL-18 mRNA expression was increased in UC from affected (*P* = .002) and unaffected areas (*P* < .001), whereas only a significant increase was found in CD from affected areas (*P* = .026). At the protein level (Figure 4), the active form of IL-18 was found expressed in most samples from UC (22 out of 26 in affected areas, 18 out of 22 in unaffected areas) and CD (12 out of 16 in affected areas, 13 out of 15 in unaffected areas), and only sporadically in healthy controls (2 out of 16). IL-23(p19) mRNA levels were increased in both affected and unaffected areas of CD (*P* = .012 and *P* = .020) and remained unmodified in UC.

### 3.3. The Highest Levels of Cytokines (IL-1*β*, IL-4, IL-6, IL-12, and IL-27) Are Found in Affected Areas and They Correlate with Markers of Tissue Damage

**Figure 3:**
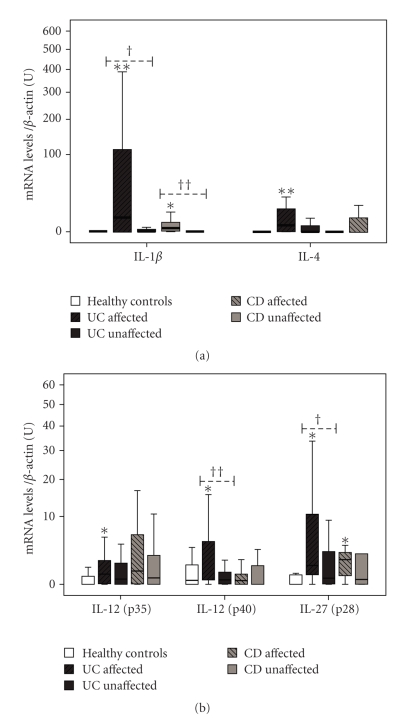
Differences in mRNA levels of those proinflammatory cytokines which are increased only in the affected areas of IBD. Statistical differences compared to healthy controls *P < .05 and **P < .001, and between affected and unaffected areas from the same pathology ^†^ *P* < .05 ^††^ *P* < .001. U: arbitrary units.

**Figure 4:**
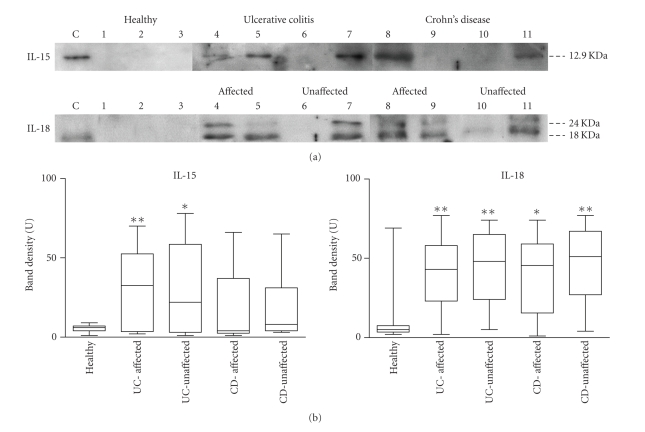
Western-blot analysis of IL-15 and IL-18 in protein extracts from intestinal biopsies. (a) Representative figure of several experiments, analysis of IL-15 (12.9 kDa), IL-18 active form (18 kDa), and IL-18 inactive form (24 kDa). Control lanes “C”: human recombinant IL-15 or IL-18. Lanes 1–3: healthy controls. Lanes 4-5: affected areas from UC. Lanes 6-7: unaffected areas from UC. Lanes 8-9: affected areas from CD. Lanes 10-11: unaffected areas from CD. (b) Densitometric band analysis, statistical differences respect to healthy controls are shown. **P < .05 and *P < .01.

IL-1*β* mRNA levels are increased in affected areas from both UC and CD, above healthy controls (*P* = .009 and *P* = .017, resp.), but also above unaffected tissue areas (*P* = .008 and *P* = .004) (Figure 3). IL-4 mRNA levels are higher in affected areas from UC, than in healthy controls (*P* = .009), whereas differences between affected and unaffected areas are close to statistical significance (*P* = .056). No changes were found when CD samples were compared. Although IL-6 is increased in all IBD groups (shown above), its levels are highest in affected areas from UC (*P* = .015, versus unaffected), with a 120-fold increase when compared to healthy controls, and ranging from 6- to 18-fold in other groups (Figure 2). mRNA levels from both subunits of IL-12, p35 and p40, have been analyzed. IL-12(p40) is increased in affected areas of UC above healthy controls (*P* = .023) and also above unaffected areas (*P* = .015), however, no changes in IL-12(p40) were observed in CD tissue samples (Figure 3). However, mRNA levels of the p35 subunit only were increased in the affected areas of UC (*P* = .044). Levels of IL-27(p28) were increased in affected areas of UC above healthy controls (*P* = .010) and also above unaffected areas of UC (*P* = .044), whereas affected areas of CD also showed increased levels of IL-27 above healthy controls (*P* = .027). 

There are significant correlations among levels of these cytokines (IL-1*β*, IL-4, IL-6, IL-12(p35 and p40), and IL-27) and those of mediators of tissue injury (iNOS and GZMB) in affected areas from UC (Table 4), whereas differences were not significant in affected areas from CD (data not shown). When comparing cytokine levels between UC and CD, only IL-12(p40) and IL-15 were found to be increased in affected areas of UC compared to affected CD (*P* = .002 and *P* = .026, resp.). 

### 3.4. Levels of SOCS-2 and SOCS-3, but Not SOCS-4 and SOCS-5, Are Increased in IBD

SOCS-2 mRNA levels are increased, above healthy controls, in both affected and unaffected areas of UC (*P* = .003 and *P* = .041), but no changes are observed in samples from CD (Figure 1). SOCS-3 levels were found upregulated in the affected areas of both UC and CD (*P* < .001 and *P* < .001), and also in the unaffected areas of UC (*P* = .027), though to a lesser extent as compared to affected areas from UC samples (*P* < .001). No statistical differences in mRNA levels of SOCS-4 and SOCS-5 were found among the different groups (data not shown). Although the analysis of SOCS-1 was part of our initial experimental design, the results were not conclusive, probably due to technical reasons.

Levels of regulatory cytokines TGF*β* and IL-10 were also analyzed. No differences were found in TGF*β* levels when study groups were compared, neither at the mRNA, nor at the protein level, measured by ELISA. No increase in the IL-10 mRNA levels was found between groups (data not shown).

## 4. Discussion

Inflammatory bowel disease (IBD) is thought to be caused by an uncontrolled response to components of the intestinal flora, which may affect individuals with genetic susceptibility together with other environmental factors [15]. The immune activation in affected tissue areas initiates the pathological process by activation of different cell types of the immune system, induction of cell infiltrate and increased apoptosis levels altogether produce major alterations in the tissue architecture of the intestine [3].

Our results confirm that affected areas from UC and CD display a high expression pattern of proinflammatory cytokines, including IFN*γ*, TNF*α*, IL-1*β*, IL4, IL-6, IL-12, IL-15, IL-18, IL-27 (Figures 2  and 3) and also molecules directly related with tissue injury, such as iNOS, GZMB, and MMP-3 (Figure 1). Despite the wide range of upregulated cytokines, only IL-1*β*, IL-4, IL-6, and IL-12 showed positive correlation with the levels of iNOS and GZMB at their mRNA levels (Table 4). These results are in accordance with other reports suggesting that IL-1*β* and IL-6 are directly involved in the regulation of mucosal damage, in both UC and CD [16], and also in animal models [17].

On the other hand, overexpression of proinflammatory immune mediators is not an exclusive feature of areas with histological alterations, previous studies have shown that the unaffected areas can also display a proinflammatory environment [18]. It has been suggested that CD is featured by an increased degree of immune activation affecting wide areas of the intestine, while those showing pathological alterations correspond to specific intestinal areas where the mechanisms of mucosal damage are triggered [4]. We can define a group of cytokines (IFN*γ*, TNF*α*, IL-6, IL-15, IL-18, and IL-23) characterized by increased levels of expression (above healthy controls) in the affected areas and, moreover, their expression is also increased in areas without histological alterations. However, the presence of this cytokines in the unaffected areas may not be sufficient to trigger mechanisms of mucosal damage. In fact, increased levels of TNF*α* [19] and IL-15 [20] have been previously reported in intestinal biopsies from IBD patients in remission without biopsy alterations. The implication of IFN*γ* and TNF*α* in the pathogenesis of IBD has been well stablished, however, they may also require the presence of other cytokines, such as IL-12, in order to activate the whole array of functions that induce mucosal damage displayed in the affected areas. 

Once the release of immune mediators in the unaffected areas has been initiated, the question of why these areas do not display histological alterations should be addressed. The SOCS-mediated intracellular suppressor activity exerts a crucial negative feedback of the cytokine signaling. SOCS-1 participates in the control of IFN*γ* responses in the affected areas from IBD [12], however we have been unable to analyze its expression levels in the unaffected areas from IBD, in a context where the involvement of the STAT1 pathway is strongly suggested by the presence of IFN*γ*. We have found that SOCS-2 is upregulated in both affected and unaffected areas from UC, but not CD (Figure 1). The biological activity of SOCS-2 has been closely related to cell growth and regulation of embryonic development in animal models, but its connection with cytokine signaling is not clear, and a more detailed study is required to define the relevance of SOCS-2 in IBD pathogenesis. Levels of SOCS-3 are strongly upregulated in the affected areas from UC and CD, and to a lesser extent in the unaffected ones from UC. This expression pattern seems to overlap with the observed for IL-6, despite the lack of correlation between its mRNA levels, and this suggests the implication of the STAT-3 signaling pathway in the pathogenesis of IBD, with IL-6 acting as activator and SOCS-3 as negative regulator [21]. Taken together, these results suggest that SOCS is not the factors that might prevent the immune activation in the unaffected tissue areas from being turned into a harmful response.

Other cytokines with regulatory function may have a role in the homeostatic mechanisms controlling mucosal damage. Previous studies have found TGF*β* to be involved during periods of active disease [22]. However, we did not find differences in TGF*β* expression (in both mRNA and protein levels), and this may be related to the high expression of Smad7 reported by others in these patients [22]. Another anti-inflammatory cytokine, IL-10, was previously found to be abundantly expressed by macrophages in areas of dense inflammatory infiltrate, and it has been directly related with the attenuation of the mucosal inflammation [16]. Contrary to other studies, our results show a decrease in the mRNA levels of IL-10 in the affected areas of UC. We believe that further studies focused on the counter-inflammatory side of immune regulation, including a broader range of regulatory cytokines and functional studies in animal models, are crucial to fully understand the connection between regulatory cytokines and the mechanisms of mucosal damage in both affected and unaffected areas of the intestine.

UC and CD are thought to share many mediators that participate in the induction of mucosal damage [3]. When comparing the gene expression profiles between these two pathologies, microarray analysis revealed overlapping patterns of upregulated genes that belong to the same functional groups [23]. However relevant differences in the molecular mechanisms that trigger them probably exist. Our results show that the affected areas from UC and CD show a similar cytokine profile, sharing most of the upregulated cytokines. We have confirmed the presence of IL-4 in UC but not in CD, a distinctive feature previously described [2]. Moreover, the presence of IL-23(p19) in CD alone might represent a hallmark of the disease, however, IL-23 shares the p40 subunit with other members of the IL-12 family making it necessary further studies to fully characterize their different roles in the pathogenesis of IBD. On the other hand, we found out that the expression levels of iNOS, GZMB, and MMP-3 in intestinal biopsies are higher in UC than in CD. This probably reflects that a higher rate of tissue damage is taking place in the affected areas from UC, and it might explain that, in most cases, the clinical outcome is faster in the later, as compared to CD. Another possible explanation is that mucosal biopsies are taken from the upper layers of the intestine, the tissue compartment in which the inflammatory activity concentrates in UC, while in CD, the inflammatory process affects to the whole tissue section of the intestine, including deeper tissue layers.

In conclusion, a great range of proinflammatory cytokines is upregulated in affected areas from IBD, but only some of them might be directly involved in triggering mechanisms of mucosal damage, whereas other cytokines might have immune-activating roles that can also be exerted beyond the affected areas of the intestine. These differential roles should be crucial to design strategies and to define targets for therapeutic interventions in UC and CD. Additionally, we believe that further studies focused on the counter-inflammatory side of immune regulation are crucial to fully understand the pathogenesis of IBD.

## Figures and Tables

**Table 1:** Clinical information from IBD patients included in the study according to the Montreal classification [13]. Age groups: A2 17–40 years, and A3 > 40 years. UC affected area: E1 proctitis, E2 left colitis or rectosigmoditis (E2-RS), and E3 pancolitis. UC grade: S1 mild colitis, S2 moderate colitis, and S3 severe colitis. CD affected area: L1 terminal ileon, L2 colon, L3 ileocolonic, and “p” periananal affectation. CD presentation: B1 nonstricturing and nonpenetrating (formerly known as inflammatory), B2 stricturing, and B3 penetrating.

Ulcerative colitis							
Case No.	Age group	Sex	Biopsy availability (affected/unnafected areas)	UC grade	Affected area	Treatment	

UC-1	A3	M	Both	S1	E2-RS	5-ASA	
UC-2	A2	M	Both	S3	E2-RS	None	
UC-3	A2	M	Both	S3	E2	None	
UC-4	A3	M	Both	S1	E2	5-ASA	
UC-5	A3	M	Both	S3	E2	None	
UC-6	A3	M	Both	S2	E1	None	
UC-7	A2	F	Both	S3	E2	None	
UC-8	A3	M	Both	S3	E1	5-ASA	
UC-9	A2	M	Both	S2	E1	None	
UC-10	A3	M	Both	S1	E1	5-ASA	
UC-11	A2	M	Both	S2	E3	Methylprednisone	
UC-12	A3	M	Both	S3	E2	None	
UC-13	A2	M	Both	S2	E1	5-ASA	
UC-14	A2	M	Both	S1	E2-RS	None	
UC-15	A3	M	Both	S2	E2	Methylprednisone	
UC-16	A3	F	Both	S3	E1	5-ASA	
UC-17	A2	M	Both	S3	E1	5-ASA	
UC-18	A3	M	Both	S3	E3	5-ASA	
UC-19	A2	M	Both	S2	E2	5-ASA	
UC-20	A2	F	Both	S3	E1	None	
UC-21	A3	M	Both	S3	E2	5-ASA	
UC-22	A3	F	Both	S1	E2-RS	None	
UC-23	A2	F	Affec. only	S3	E3	5-ASA	
UC-24	A2	F	Affec. only	S2	E2	5-ASA	
UC-25	A3	M	Affec. only	S2	E2	5-ASA	
UC-26	A3	M	Affec. only	S2	E1	5-ASA	

Crohn's disease							

Case No.	Age group	Sex	Biopsy availability (affected/unnafected areas)	CD presentation	Affected area	Surgery	Treatment

CD-1	A2	M	Both	B1	L1	No	None
CD-2	A3	F	Both	B2	L1	No	None
CD-3	A3	M	Both	B3	L2	Yes	Budesonide
CD-4	A2	F	Both	B1p	L1	Yes	Topic steroids + 5-ASA
CD-5	A2	M	Both	B1	L1	No	Oral steroids
CD-6	A2	M	Both	B1	L2	No	5-ASA
CD-7	A2	M	Both	B3	L3	Yes	Budesonide + 5-ASA
CD-8	A3	F	Both	B1p	L1	Yes	Topic steroids + 5-ASA
CD-9	A2	F	Both	B2	L1	No	None
CD-10	A2	F	Both	B1	L1	No	5-ASA
CD-11	A2	F	Both	B1	L1	No	5-ASA
CD-12	A3	M	Both	B3	L1	No	5-ASA
CD-13	A2	F	Both	B1	L1	No	5-ASA
CD-14	A2	F	Both	B1	L1	No	None
CD-15	A2	M	Both	B1p	L3	Yes	Topic 5-ASA
CD-16	A3	M	Affec. only	B1	L1	No	5-ASA

**Table 2:** Real-time PCR information. Primer sequences, PCR products length, temperature of annealing, and source of the primers used for Quantitative PCR.

Gene	Forward primer (5′-3′)	Reverse primer (5′-3′)	PCR product length (base pairs)	Annealing temperature	Primer source
*β*-actin	atg ggt cag aag gat tcc tat gtg	ctt cat gag gta gtc agt cag gtc	359	60	M. Bongers, E. Liehl, J. Barsig, Focus 21, 66 (2006)
GZMB	aag acg act tcg tgc tga ca	ccc caa ggt gac att tat gg	62	60	Universal Probe Library Human#60 (Roche)
IFN*γ*	tgg aaa gag gag agt gac ag	att cat gtc ttc ctt gat gg	129	60	M. G. Karlsson, J. Ludvigsson, Diabetes Res. Clin. Pract. 40, 21 (1998)
IL-1*β*	tcc gac cac cac tac ag	cgg agc gtg cag ttc a	224	53	Designed with the software LightCycler Probe Design 2 (Roche)
IL-4	ttc tac agc cac cat gag	cat gat cgt ctt tag cct ttc	198	59	Designed with the software LightCycler Probe Design 2 (Roche)
IL-6	Commercial reagent		—	60	TaqMan Gene Expression Assay Hs00174131_ml (Applied Biosystems)
IL-10	Commercial reagent		—	60	TaqMan Gene Expression Assay Hs00174086_ml (Applied Biosystems)
IL-12(p35)	tgt cac cga gaa gct gat gt	gag gtt tct ggc caa act ga	278	68	Designed with the software LightCycler Probe Design 2 (Roche)
IL-12(p40)	Commercial reagent		—	60	TaqMan Gene Expression Assay Hs00233688_ml (Applied Biosystems)
IL-15	tgt ctt cat ttt ggg ctg ttt ca	tcc tcc agt tcc tca cat tct ttg	327	60	C. Kebelmann-Betzing et al., Cytokine 13, 39 (2001)
IL-18	gcttga atc taa att atc agt c	caa att gca tct tat tat cat g	335	55	T. Tomita et al., J. Infect. Dis. 183, 620 (2001)
IL-23(p19)	agc agc tca agg atg gca ctc ag	ccc caa att tcc ctt ccc atc ta	251	55	A. Wesa et al, BMC Immunol. 3, 14 (2002)
IL-27(p28)	gcg gaa tct cac ctg cca	gga aac atc agg gag ctg ctc	69	64	S. Pflanz et al, Immunity 16, 779 (2002)
iNOS	tct gca gac acg tgc gtt act	atg cac agc tga gca ttc ca	115	56	M. Ohtsuki et al., Clin. Chim. Acta 353, 103 (2005)
MMP-3	gaa atg cag aag ttc ctt gg	gtg aaa gag acc cag gga gtg	489	60	T. Sadowski, J. Steinmeyer, Inflamm. Res. 50, 175 (2001)
SOCS-2	agt gtg gtt cat ctg atc g	aca ttt gtt aat ggt gag cct	162	54	Designed with the software LightCycler Probe Design 2 (Roche)
SOCS-3	ggc cac tct tca gca tct c	atc gta ctg gtc cag gaa ctc	109	62	N. Torpey et al, J. Biol. Chem. 279, 26789 (2004)
SOCS-4	cct atg act ggc tct gt	gct tcg gct gcg tat t	269	52	Designed with the software LightCycler Probe Design 2 (Roche)
SOCS-5	cct aca ggt gtt cag taa gac	cca cac tgt tga aat act cat cc	171	56	Designed with the software LightCycler Probe Design 2 (Roche)
TGF*β*_1_	gga cac caa cta ttg ctt cag	tcc agg ctc caa atg tag g	148	60	W. X. Chen et al., World J. Gastroenterol. 8, 379 (2002)
TIMP-1	Commercial reagent		—	60	TaqMan Gene Expression Assay Hs00171558_ml (Applied Biosystems)
TNF*α*	tca gat cat ctt ctc gaa cc	cag ata gat ggg ctc ata cc	361	60	G. J. Atkins et al., Bone 26, 653 (2000)

**Table 3:** Overview of mRNA expression in intestinal biopsies from UC and CD patients. ↑, ↓, and =: increased, decreased, or unchanged mRNA levels respect to healthy controls, Mann-Whitney *P* < .05.

	Ulcerative colitis		Crohn's disease	
	Affected	Unaffected	Affected	Unaffected
Mediators of mucosal damage				

GZMB	↑	=	↑	=
iNOS	↑	=	↑	=
MMP-3	↑	=	=	=
TIMP-1	↑	=	↑	=

Proinflammatory cytokines in affected areas only				

IL-1*β*	↑	=	↑	=
IL-4	↑	=	=	=
IL-12(p35)	↑	=	↑	=
IL-12(p40)	↑	=	=	=
IL-27(p28)	↑	=	↑	=

Cytokines in affected and unaffected areas				

IFN*γ*	↑	↑	↑	↑
TNF*α*	↑	↑	↑	↑
IL-6	↑	↑	↑	↑
IL-15	WB ↑/mRNA ↑	WB ↑/mRNA ↑	WB ↑/mRNA =	WB ↑/mRNA =
IL-18	WB ↑/mRNA ↑	WB ↑/mRNA ↑	WB ↑/mRNA ↑	WB ↑/mRNA =
IL-23(p19)	=	↑	↑	↑

SOCS family				

SOCS-2	↑	=	=	=
SOCS-3	↑	↑	↑	=
SOCS-4	↑	=	=	=
SOCS-5	=	=	=	=

Regulatory cytokines				

IL-10	=	↓	=	=
TGF*β*_1_	=	=	=	=

**Table 4:** Correlation analysis of mRNA levels among cytokines and effector molecules in affected areas from UC (Spearman's correlation rank).

	iNOS	Granzyme B	MMP-3
Proinflammatory cytokines in affected areas only			

IL-1*β*	*P* < .001	*P* < .001	N.S.
IL-4	N.S.	*P* = .048	N.S.
IL-12(p35)	*P* = .011	*P* = .018	N.S.
IL-12(p40)	*P* = .039	*P* = .020	N.S.
IL-27(p28)	*P* = .037	N.S.	N.S.

Cytokines in affected and unaffected areas			

IFN*γ*	N.S.	N.S.	N.S.
TNF*α*	N.S.	N.S.	N.S.
IL-6	*P* = .001	*P* = .003	N.S.
IL-15	N.S.	*P* = .021	N.S.
IL-18	N.S.	N.S.	N.S.
IL-23(p19)	N.S.	N.S.	N.S.
